# Combination and Differentiation Theories of Categorization: A Comparison Using Participants’ Categorization Descriptions

**DOI:** 10.1162/opmi_a_00187

**Published:** 2025-02-08

**Authors:** Sujith Thomas, Aditya Kapoor, Narayanan Srinivasan

**Affiliations:** Department of Computer Science & Information Systems, BITS Pilani, K. K. Birla Goa Campus, Goa, India; APPCAIR, BITS Pilani, K. K. Birla Goa Campus, Goa, India; Department of Cognitive Science, Indian Institute of Technology Kanpur, Kanpur, India

**Keywords:** supervised category learning, unidimensional categorization, classification learning, multidimensional categorization, combination theory

## Abstract

Differentiation and Combination theories make different predictions about the order in which information is processed during categorization. Differentiation theory posits that holistic processing of a stimulus occurs before individual features are processed. According to Differentiation theory, overall similarity-based categorization is faster and less effortful compared to unidimensional categorization. In contrast, Combination theory posits that individual features are processed first and that information regarding these features must be combined during multidimensional categorization. According to Combination theory, overall similarity-based categorization is more effortful and takes more time compared to unidimensional categorization. In this study, we trained participants to learn artificial categories using classification learning and observation learning procedures. We used participants’ categorization descriptions to determine the number of stimuli dimensions used for categorization. Our results from the first three experiments show that participants who used more dimensions took more time to categorize the transfer stimuli, consistent with Combination theory. In Experiment 4, we tested the hypothesis that using more dimensions takes more time solely due to multiple eye fixations and saccades. In our study, we used visual stimuli with features that do not overlap in space. Our results show that while performing a multidimensional task, participants need more time to recall the feature-category associations learned during the experiment, making the task more effortful, as predicted by Combination theory. Further studies are needed to determine whether Combination theory applies to other types of stimuli, particularly those with spatially non-separable features.

## INTRODUCTION

Studies that use a variety of classification tasks have shown that humans prefer different categorization strategies depending on the presence of time pressure and cognitive load (Milton et al., 2008; Smith & Shapiro, 1989; Ward, 1983; Wills et al., 2013). These studies disagree on whether overall similarity-based categorization takes less time (is less effortful) or more time (is more effortful) compared to a simple (unidimensional) categorization strategy.

Early studies using the triad task reported that overall similarity-based sorting required less resource and less time compared to unidimensional sorting (Smith & Kemler Nelson, 1984; Smith & Shapiro, 1989; Ward, 1983). Holistic processing, which involves using all features, is a fundamental and primitive aspect of human cognition. It occurs at an early stage of cognitive processing, prior to the accessibility of individual stimulus dimensions (Smith & Kemler Nelson, 1984). In this mode of processing, individuals often make categorization decisions based on a broad, “first-impression” assessment. This involves perceiving stimuli as undifferentiated wholes or “blobs” (Smith & Shapiro, 1989). Holistic processing is facilitated by a *preattentive* mechanism at the early stage of processing, which requires fewer cognitive resources (Smith & Kemler Nelson, 1984; Smith & Shapiro, 1989).

These studies support Differentiation theory, which posits that a stimulus appears as an undifferentiated whole (preattentive holistic processing occurs first), and a simple categorization strategy involves *differentiating* a stimulus into its constituent features, which takes more time (Brooks, 1978; Ward, 1983; Wills et al., 2015).

However, there are studies that do not support Differentiation theory (Wills et al., 2013, 2015). Studies that used the match-to-standards procedure showed that multidimensional strategies are more effortful and take more time (Milton et al., 2008; Wills et al., 2013). The results from the match-to-standards procedure support Combination theory, where information regarding constituent features is processed first, and a multidimensional strategy involves a *combination* of feature-level information, which is more effortful and takes more time (Wills et al., 2013, 2015).

Wills et al. (2015) used response-set analysis, which, unlike traditional analysis, takes into account whether participant responses can be explained by a unidimensional strategy based on a single non-criterial/non-identity dimension. Response-set analysis revealed that support for Differentiation theory could arise because a unidimensional strategy can be misidentified as an overall similarity-based strategy. Although response-set analysis considers more possible explanations for each participant’s responses, its ability to distinguish between categorization strategies depends on the stimuli set used (Wills et al., 2015). For example, using response-set analysis, it can be difficult to distinguish between a perfectly accurate non-criterial-attribute-based unidimensional categorization and a less accurate overall similarity-based categorization.

In this study, we used participants’ categorization descriptions to determine the number of stimuli dimensions used by each participant during categorization. Combination theory predicts that participants who use more stimuli dimensions will take more time to categorize, whereas Differentiation theory predicts the opposite. We wanted to determine which theory better explains categorization behavior after a categorization strategy is learned. Wills et al. (2015) reported that overall similarity-based categorization is rarely used in both classification and observation learning procedures. In this study, we also aimed to replicate this finding.

In Experiment 1, we used both classification and observation learning in the training phase. We expected the categorization behavior in the transfer phase to support Combination theory. In Experiment 2, we modified our stimuli so that colors could serve as memory cues for feature diagnosticity. We wanted to test whether holistic processing of stimuli would become faster in the presence of color cues, thereby providing evidence in favor of Differentiation theory. In Experiment 3, we repeated Experiment 1 without using observation learning in the training phase. We expected the results of Experiment 3 to be similar to those of Experiment 1, supporting Combination theory.

In Experiment 4, we tested the hypothesis that the additional time taken when using more stimuli dimensions could be explained purely by the time required for eye fixations and saccades. We expected the results to show that the addtional time taken when using more dimensions is also due to the time required to recall feature-category associations learned during the experiment, which would further support Combination theory.

In the rest of this article, we refer to the perfectly diagnostic stimuli dimension as the CA (criterion attribute) dimension and the partially diagnostic dimensions as the FR (family resemblance) dimensions.

## EXPERIMENT 1

In Experiment 1, we used both classification learning and observation learning blocks in the training phase. Wills et al. (2015) showed that overall similarity-based categorization is rare in both classification and observation learning procedures. Additionally, the Combination/Differentiation theory makes a general claim about whether cognition during categorization begins with an undifferentiated stimulus whole or with the stimulus attributes, regardless of the categorization task (Wills et al., 2015, 2020). For these reasons, we felt that using both classification and observation learning in the training phase would not introduce a confound.

In Experiment 1, we aimed to determine which theory—Combination or Differentiation—would be better supported by the categorization behavior in the transfer phase after the learning criterion was achieved. We used participants’ categorization strategy descriptions to determine the number of stimuli dimensions used by each participant. We expected participants who used more dimensions to have a higher mean response time in the transfer phase, which would support Combination theory.

**Figure 1.**
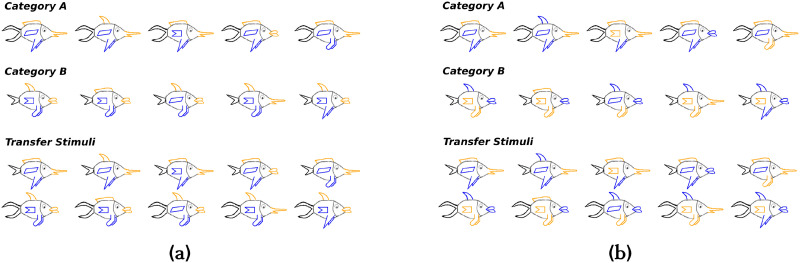
(a) Training and transfer stimuli used in Experiment 1 (one of the five sets). In the stimuli set shown, the shape of the tail is the perfectly diagnostic (CA) dimension, and the remaining features are partially diagnostic (FR) features. In Experiment 1, colours do not help in remembering the diagnosticity of FR features. (b) Training and transfer stimuli used in Experiment 2 (one of the five sets). In Experiment 2, colours covary with the FR features and can serve as memory cues to remember feature diagnosticity.

Experiments 1, 2, and 3 used stimuli sets where the FR features are colored. In Experiment 1, the colors of the FR features did not aid in remembering feature diagnosticity. However, in Experiment 2, the colors covaried with the FR features and could help in remembering feature diagnosticity (see Figure 1).

### Methods

#### Subjects.

Forty five volunteers (females = 5; males = 40; mean age = 21.5 years) participated in this experiment. All the participants were undergraduate students. The linear regression results reported in a previous study indicated that there was an effect between the accuracy for FR features and percentage of unidimensional categorization (*R*^2^ = .16 in condition M0) (Thomas & Srinivasan, 2023). Power analysis (Bausell & Li, 2002) indicated 44 subjects are needed for power = .80, *R*^2^ = .16 and two-tailed *α* = .05 for a significant effect between the accuracy for FR features and percentage of unidimensional categorization. This study was approved by the Human Ethical Committee of our institute. Participants read a soft copy of the information document and gave their consent by clicking on a checkbox before the start of the online experiment.

#### Materials.

Figure 1a shows the fish-like stimuli that were used in Experiment 1. The stimuli were designed based on the stimuli used by Rabi et al. (2015). The stimuli consisted of five dimensions—shape of the mouth, shape of the upper-fin, shape of the lower-fin, body pattern and shape of the tail. Each stimuli dimension could take one of two possible values. One stimuli dimension was perfectly diagnostic of category membership (CA dimension), and the remaining four dimensions were partially diagnostic (FR dimensions). In Figure 1a, the shape of the tail forms the CA dimension. The top two rows of Figure 1a show the training stimuli used in Experiment 1, and the bottom two rows show the transfer stimuli. The transfer stimuli were constructed by flipping the CA feature of the training stimuli. For example, the transfer stimuli shown in the third row are just like the training stimuli shown in the first row, except that the CA feature has been flipped. So, the transfer stimuli contained the CA feature of one category and the FR features of the opposite category.

We used five sets of stimuli, where in each set a different stimuli dimension formed the CA dimension. Figure 1a shows one of the five sets that were used in Experiment 1. Each participant did the experiment using one of the five sets of stimuli. In the experiment, each of the five sets of stimuli was used 9 times (9 × 5 = 45 participants). In each of the five sets of stimuli, the CA dimension was always black in colour, two FR dimensions were always blue, and the remaining two FR dimensions were always yellow. The colours on their own did not help in identifying the categories (as can be see in Figure 1a). Colours can make the FR dimensions more salient, but a pilot study revealed that participants continued to show a strong preference for the CA dimension based strategy. In Experiment 2, we have used stimuli in which colours covary with the FR features (shown in Figure 1b). The stimuli sets are available publicly at OSF: https://osf.io/fkqcy/.

#### Procedure.

The behavioural data for the experiment was collected using a web application. The link to the web application was sent to participants over email. Participants responded in a self-paced manner in all the trials throughout the experiment. As mentioned earlier, each participant did the experiment using one of the five sets of stimuli.

The experiment started with the training phase. Participants were told that they had to learn to differentiate between category A objects and category B objects. Participants were given neutral instructions that did not indicate the categorization strategy they were expected to use. Each block of the training phase contained two sub-blocks. In the first sub-block, participants learned the categories using observation learning. The training stimuli were presented one by one along with its correct category label. In each trial, participants were expected to press the same key as the category label (i.e., key A was pressed for category label A and key B for category label B). Each stimulus was presented for the entire duration of the trial and each trial ended when the participant responded with a key press. In the second sub-block, participants learned the categories using classification learning. The training stimuli were again presented one by one. Participants had to categorize each training stimulus by pressing either A or B on the keyboard. Again, each stimulus was presented for the entire duration of the trial and each trial ended when the participant responded with a key press.

After each trial in the classification sub-block, feedback was provided which indicated whether the categorization response was correct. The feedback appeared on the screen for one second. At the end of the classification learning sub-block, participants were shown their accuracy for the ten training stimuli. Participants had to achieve an accuracy of 90% twice (learning criterion) in order to proceed to the transfer phase. The training blocks were repeated until participants could achieve an accuracy of 90% two times. In both the sub-blocks of the training phase, the ten training stimuli were presented in a random order.

The training phase was followed by the transfer phase. In each block of the transfer phase, all the ten transfer stimuli were presented one by one in a random order. Participants categorized each transfer stimulus by pressing either A or B. Each trial was response terminated and the stimulus appeared on the screen during the entire trial. No feedback was given at the end of trials. There were three blocks in the transfer phase. This means that participants categorized each transfer stimulus three times in the transfer phase.

The transfer phase was followed by an all features test phase. Participants were not informed that all the features will be tested in the experiment. The stimuli take two features along each of the five dimensions. So, there were 10 features in total. In each block of the all features test phase, participants were shown all the 10 features one by one in a random order. In each trial, participants were asked to identify the category in which a given feature occurred more commonly. Participants could respond by pressing either A or B key. Each trial was response terminated. No feedback was given at the end of trials. There were three blocks in the all features test phase.

After the all features test phase, participants were asked to describe the categorization strategy that they used. Participants were requested to describe their strategy with sufficient clarity so that another person may read the description and replicate the categorization pattern.

### Results and Discussion

Two independent raters read the categorization descriptions for all the three experiments in this study. The raters determined the number of stimuli dimensions used for categorization from each description. The two raters were blind to the hypotheses being tested. For each categorization description, the raters either selected a number from 1 to 5 or marked the description as “Incomprehensible”.

The raters’ ratings were the same for 64.4% of the descriptions in Experiment 1. For six descriptions (out of 45), at least one of the raters marked the description as incomprehensible. We have not used the data for these six (incomprehensible) participants for obtaining the results of the statistical analyses given below. For the remaining 39 descriptions, the mean absolute difference between the ratings was .49, which means that the two ratings were very close on an average. The inter-rater reliability was found to be Cohen’s *κ* = .57 (moderate agreement). We have found the average rating of the two raters for the 39 (comprehensible) participants. The average rating was considered to be the number of dimensions used by each of the 39 participants.

Table 1 shows an overall summary. Twenty participants used less than 2 dimensions for categorization, and 5 participants used four or more dimensions for categorization. Table 1 also shows that participants who used less than two dimensions responded faster compared to the other groups. Since some of the data was not normally distributed, we performed Spearman’s rank correlation; however, we do provide linear regression results as well in the footnotes. Spearman’s rank correlation between the mean response time in the transfer phase and the number of stimuli dimensions used was positive, *r*(37) = .60, *p* < .0001.^1^ Participants who used more stimuli dimensions took more time to respond in the transfer phase. This result supports the Combination theory.

**Table 1.** The four columns represent the groups based on the number of stimuli dimensions used by the participants. The number of dimensions used was the mean rating given by the two raters based on each participant’s description. The rows show the number of participants in each group (row 1), mean response time for categorizing the transfer stimuli (row 2), mean accuracy for the stimuli dimensions in the all features test phase (row 3), average number of dimensions for which a participant achieved 100% accuracy in the all features test phase (row 4), mean response time for classification learning trials in the training phase (row 5) and mean number of training blocks needed to achieve the learning criterion (row 6). I.D. refers to Incomprehensible Description.

Dim. Used (*x*)	*x* < 2	2 ≤ *x* < 4	4 ≤ *x* ≤ 5	I.D.
1. No. of participants	20	14	5	6
2. Mean RT (transfer)	1.48 s	3.37 s	7.14 s	4.91 s
3. Dim. accuracy	75.67%	81.90%	91.33%	75.56%
4. Dim. 100% accuracy	2.3	2.93	4.2	2.17
5. Mean RT (training)	1.87 s	4.18 s	5.28 s	4.87 s
6. Training blocks	2.35	2.79	2.60	4.33

Exploratory analysis revealed that participants who used more stimuli dimensions learned the diagnostic features of each category better. Spearman’s rank correlation between the overall accuracy in the all-features test phase and the number of stimuli dimensions used was positive, *r*(37) = .39, *p* = .01.^1^

Due to this result, we wanted to check whether a stimuli dimension is used only when it is learned with some minimum level of accuracy. We used Bayesian modeling to find the minimum accuracy with which a stimuli dimension must be learned for it to be used for categorization (see the Supplemental material). The Bayesian model (see Figure 1 in the Supplemental material) predicts the probability that a participant will categorize a stimulus as belonging to category A. The key feature of the Bayesian model is the use of a step function that connects how well a stimuli dimension is learned with whether the dimension is used for categorization. The results of Bayesian modeling show that a stimuli dimension must be learned with a high level of accuracy for it to be used for categorization. We used Bayesian models corresponding to different theoretical positions. Our results show that a model that uses a partially diagnostic feature only when it is learned accurately provides a better explanation for the data. This is consistent with the results reported in a previous study (Thomas & Srinivasan, 2023).

If a participant does not make any mistakes in the all-features test phase for a particular stimuli dimension, then we say that the participant learned the dimension with 100% accuracy. We found the number of stimuli dimensions each participant learned with 100% accuracy. Spearman’s rank correlation between the number of stimuli dimensions learned with 100% accuracy and the number of stimuli dimensions used was positive, *r*(37) = .43, *p* = .006.^1^ This result is consistent with the results of Bayesian modeling.

We also performed an exploratory analysis of the data from the training phase of the experiment to check whether the Combination theory would also apply to the learning phase of the experiment. More specifically, we wanted to check whether participants who used more stimuli dimensions performed worse in the training phase of the experiment. The training phase included both observation learning and classification learning trials. Unfortunately, we did not record the response time for the observation learning trials in the training phase; however, we did record the response time for the classification learning trials. Table 1 shows that participants who used more stimuli dimensions took more time to respond to classification learning trials during the training phase of Experiment 1. Spearman’s rank correlation between the mean response time in classification learning during the training phase and the number of stimuli dimensions used for the categorization task was positive, *r*(37) = .54, *p* < .001. Further studies are needed to establish the connection between Combination theory and how different categorization strategies are learned.

Table 1 shows the number of training blocks needed to achieve the learning criterion. We wanted to determine whether participants who used more stimuli dimensions were those who found it difficult to achieve the learning criterion. However, we could not find evidence for this. Spearman’s rank correlation between the number of training blocks and the number of stimuli dimensions used was not significant, *r*(37) = .25, *p* = .12.

Overall, our results show that participants who used more stimuli dimensions had a higher mean response time in the transfer phase. This finding is consistent with Combination theory and inconsistent with Differentiation theory. Further analysis revealed that participants who used multiple stimuli dimensions had high accuracy for multiple stimuli dimensions in the all-features test phase.

## EXPERIMENT 2

In Experiment 2, we modified the stimuli used in Experiment 1 such that the colours covary with the FR features. This allowed the colours to act as memory cues for remembering the diagnosticity of the FR features. We wanted to test whether using color cues for remembering the family resemblance structure would make holistic processing faster. If holistic processing becomes less effortful and faster in the presence of color cues, it would provide support for the Differentiation theory. If the results are similar to those in Experiment 1, then it would support the Combination theory.

### Methods

#### Subjects.

Forty five volunteers (7 females; mean age = 21.2 years) participated in this experiment. All the participants were undergraduate students. The results of linear regression reported in a previous study indicated that there was an effect between the accuracy for FR features and percentage of unidimensional categorization (*R*^2^ = .16 in condition M0) (Thomas & Srinivasan, 2023). We expected a similar effect to exist in Experiment 2. Power analysis (Bausell & Li, 2002) indicated 44 subjects are needed for power = .80, *R*^2^ = .16 and two-tailed *α* = .05 for a significant effect between the accuracy for FR features and percentage of CA categorization.

#### Materials.

Figure 1b shows the fish-like stimuli that were used in Experiment 2. The colour of an FR dimension was perfectly correlated with the FR feature. Apart from this, all the other details of the stimuli were same as that of Experiment 1. Just like in Experiment 1, we had five sets of stimuli, where a different stimuli dimension formed the CA dimension in each set. In Figure 1b, the left most item shown under category A and B formed the prototype for the respective categories. For the prototypes in each of the five stimuli sets, the CA feature was always black, two FR features were always blue and the remaining two FR features were always yellow (see the category prototypes in Figure 1b). This was done to ensure that participants cannot use a simple strategy based only on colours like “category A items have mostly yellow features.”

Figure 1b shows one of the five sets of stimuli that were used in Experiment 2. The five sets of stimuli that were used in Experiment 1 and 2 were exactly the same in terms of the shape of the features. The only difference was that in Experiment 2 colours were perfectly correlated with the FR features.

#### Procedure.

The experimental procedure in Experiment 2 was same as that in Experiment 1.

### Results and Discussion

The raters’ ratings were identical for 62.2% of the descriptions in Experiment 2. For nine descriptions (out of 45), at least one of the raters marked the description as incomprehensible. We have not used the data for these nine participants for obtaining the results of the statistical analyses given below. For the remaining 36 descriptions, the mean absolute difference between the ratings was .44, which means that the two ratings were very close on an average. The inter-rater reliability was found to be Cohen’s *κ* = .55 (moderate agreement). We have found the average rating of the two raters for the 36 (comprehensible) participants. The average rating was considered to be the number of dimensions used by each of the 36 participants.

Table 2 shows that very few participants performed an overall similarity based categorization. The summary data also shows that participants who used fewer stimuli dimensions were faster in their categorization response. Spearman’s rank correlation between the mean response time in the transfer phase and the number of stimuli dimensions used was positive, *r*(34) = .69, *p* < .0001.^2^ Participants who used more stimuli dimensions took more time to respond in the transfer phase. This result again supports the Combination theory.

**Table 2.** The four columns represent the groups based on the number of stimuli dimensions used by the participants. The number of dimensions used was the mean rating given by the two raters based on each participant’s description. The rows show the number of participants in each group (row 1), mean response time for categorizing the transfer stimuli (row 2), mean accuracy for the stimuli dimensions in the all features test phase (row 3), average number of dimensions for which a participant achieved 100% accuracy in the all features test phase (row 4), mean response time for classification learning trials in the training phase (row 5) and mean number of training blocks needed to achieve the learning criterion (row 6). I.D. refers to Incomprehensible Description.

Dim. Used (*x*)	*x* < 2	2 ≤ *x* < 4	4 ≤ *x* ≤ 5	I.D.
1. No. of participants	17	12	7	9
2. Mean RT (transfer)	2.10 s	4.42 s	10.60 s	3.41 s
3. Dim. accuracy	70.98%	80.56%	87.14%	70.0%
4. Dim. 100% accuracy	1.94	2.58	3.86	2.00
5. Mean RT (training)	2.94 s	4.82 s	8.89 s	3.93 s
6. Training blocks	3.76	4.00	3.00	3.11

We performed further analysis to check whether participants who used more dimensions were also more accurate about feature diagnosticity across multiple dimensions. Spearman’s rank correlation between the number of stimuli dimensions learned with 100% accuracy and the number of stimuli dimensions used was positive, *r*(34) = .43, *p* = .009.^2^ Spearman’s rank correlation between the overall accuracy in the all features test phase and the number of stimuli dimensions used was also positive, *r*(34) = .36, *p* = .029.^2^

We have used the same Bayesian model used for Experiment 1 to check whether there is a minimum accuracy with which a stimuli dimension must be learned for it to be used for categorization (see the Supplemental material). The results of Bayesian modelling (see Figure 2 in the Supplemental material) show that a stimuli dimension is used only when it is learned with a high level of accuracy. We have again compared the same Bayesian models that were compared for Experiment 1. Our results show that the model that allows a partially diagnostic dimension to be used only when it is learned with a high accuracy provides a better explanation for the data. This result is consistent with the results reported in a previous study (Thomas & Srinivasan, 2023). The above results show that in Experiment 2 participants who used multiple dimensions had learned those dimensions with a high accuracy.

We performed an exploratory analysis of the data from the training phase of Experiment 2. As mentioned earlier, we recorded the response times for the classification learning trials but not the observation learning trials in the training phase of the experiment. Table 2 shows that participants who used more stimuli dimensions took more time to respond to classification learning trials during the training phase of Experiment 2. Spearman’s rank correlation between the mean response time in the classification learning subblock of the training phase and the number of stimuli dimensions used for the categorization task was positive, *r*(34) = .58, *p* < .001. This indicates that participants who used more dimensions also took more time in the classification trials of the training phase. The last row in Table 2 shows the number of training blocks needed to achieve the learning criterion. We wanted to determine whether participants who used more stimuli dimensions were those who found it difficult to achieve the learning criterion. However, we could not find evidence for this. Spearman’s rank correlation between the number of training blocks and the number of stimuli dimensions used was not significant, *r*(34) = .06, *p* = .71.

Despite the presence of color cues, participants who used more dimensions took more time to categorize the transfer stimuli in Experiment 2. Our results continue to support the Combination theory. Only 7 participants performed an overall similarity based categorization in Experiment 2. This result is consistent with the finding that overall similarity based categorization is rare in both classification and observation learning procedures (Wills et al., 2015).

Our results also show that participants who prefer a multidimensional strategy also learn multiple stimuli dimensions with a high accuracy.

## EXPERIMENT 3

An anonymous reviewer suggested that using both classification and observation learning in the training phase could introduce a confound. To address this, we aimed to replicate the results of Experiment 1 without using observation learning in the training phase. In Experiment 3, we used only classification learning in the training phase and omitted observation learning blocks. This allowed us to apply the same learning criterion as used in Experiment 1. All other details of Experiment 3, including the stimuli, were the same as in Experiment 1. We expected to obtain similar results to those observed in Experiment 1.

### Methods

#### Subjects.

Fifty volunteers (females = 3; males = 47; mean age = 20.94 years) participated in this experiment. All the participants were undergraduate students. The results of linear regression reported in a previous study indicated that there was an effect between the accuracy for FR features and percentage of CA categorization (*R*^2^ = .16 in condition M0) (Thomas & Srinivasan, 2023). Power analysis (Bausell & Li, 2002) indicated 44 subjects are needed for power = .80, *R*^2^ = .16 and two-tailed *α* = .05 for a significant effect between the accuracy for FR features and percentage of CA categorization.

#### Materials.

The stimuli that were used for Experiment 3 were same as those used for Experiment 1. As described earlier, there were five sets of stimuli where each set had a different CA dimension. Figure 1a shows one of the five sets of stimuli that were used.

#### Procedure.

We made only one modification to the experimental procedure used in Experiment 1. Each block of the training phase consisted of classification learning only (i.e., there were no observation learning sub-blocks). Except for this modification, all the other details of Experiment 3 were same as that of Experiment 1.

### Results and Discussion

The raters’ ratings were identical for 66.0% of the descriptions in Experiment 3. For four descriptions (out of 50), at least one of the raters marked the description as incomprehensible. We have not used the data for these four participants for obtaining the results of the statistical analyses given below. For the remaining 46 descriptions, the mean absolute difference between the ratings was .35. The inter-rater reliability was found to be Cohen’s *κ* = .59 (moderate agreement). We found the average rating of the two raters for each of the 46 (comprehensible) participants. The average rating was considered to be the number of dimensions used by each of the 46 participants.

Table 3 shows that only 10 (out of 50) participants preferred an overall similarity based categorization. The summary data in Table 3 suggests that participants who used more stimuli dimensions had a higher mean response time in the transfer phase. Spearman’s rank correlation between the mean response time in the transfer phase and the number of stimuli dimensions used was positive, *r*(44) = .53, *p* = .0001.^3^ This indicates that the support for Combination theory in the previous two experiments was not due to the presence of observation learning in the training phase. Our results remain consistent with Combination theory.

**Table 3.** The four columns represent the groups based on the number of stimuli dimensions used by the participants. The number of dimensions used was the mean rating given by the two raters based on each participant’s description. The rows show the number of participants in each group (row 1), mean response time for categorizing the transfer stimuli (row 2), mean accuracy for the stimuli dimensions in the all features test phase (row 3), average number of dimensions for which a participant achieved 100% accuracy in the all features test phase (row 4), mean response time for classification learning trials in the training phase (row 5) and mean number of training blocks needed to achieve the learning criterion (row 6). I.D. refers to Incomprehensible Description.

Dim. Used (*x*)	*x* < 2	2 ≤ *x* < 4	4 ≤ *x* ≤ 5	I.D.
1. No. of participants	20	16	10	4
2. Mean RT (transfer)	2.41 s	3.41 s	5.34 s	7.97 s
3. Dim. accuracy	75.17%	67.29%	82.33%	81.67%
4. Dim. 100% accuracy	2.35	2.44	3.80	3.25
5. Mean RT (training)	4.28 s	3.99 s	6.49 s	8.68 s
6. Training blocks	4.15	6.25	4.80	6.00

We also checked whether the number of stimuli dimensions learned with 100% accuracy was correlated with the number of stimuli dimensions used. Spearman’s rank correlation between the number of stimuli dimensions learned with 100% accuracy and the number of stimuli dimensions used was positive, *r*(44) = .36, *p* = .01.^3^ We have also used the same Bayesian model as was used for Experiment 1 and 2 to check whether a stimuli dimensions must be learned with a minimum accuracy to be used for categorization (see the Supplemental material). Results of Bayesian modelling show that a stimuli dimension is used only when it is learned with a high level of accuracy (see Figure 2 in the Supplemental material). Additionally, our modelling results show that a model that allows a partially diagnostic dimension to be used only when it is learned with a high accuracy provides a better explanation for the data. This result is consistent with the previously reported results (Thomas & Srinivasan, 2023).

In Experiment 3, we could not find a significant correlation between the overall accuracy in the all features test phase and the number of stimuli dimensions used. Spearman’s rank correlation between the two variables was found be *r*(44) = .17, *ns*.^3^ There were participants who had a high overall accuracy in the all features test phase, but did not have 100% accuracy for any stimuli dimension. These participants reported to have used only a few stimuli dimensions. There were also participants who learned the features to category label mapping in an incorrect (opposite) manner. Such participants obtained 0% accuracy for one or two stimuli dimensions, which resulted in a decrease in their overall accuracy percentage. So, the overall accuracy of these participants were low even though they used multiple stimuli dimensions.

We performed an exploratory analysis of the data from the training phase of Experiment 3, which did not include an observation learning subblock. Table 3 shows the response times for the classification learning trials in the training phase of Experiment 3. We did not find a significant correlation (although there was trend) between the mean response time in the training phase and the number of stimuli dimensions used, *r*(44) = .25, *p* = .09. The last row in Table 3 shows the number of training blocks needed to achieve the learning criterion. We wanted to determine whether participants who used more stimuli dimensions were those who found it difficult to achieve the learning criterion. However, we could not find evidence for this. Spearman’s rank correlation between the number of training blocks and the number of stimuli dimensions used was not significant, *r*(44) = .21, *p* = .16.

Our results in Experiment 3 show that using more dimensions requires more time. The results continue to support the Combination theory, even without observation learning in the training phase. We also continue to find a correlation between the number of stimuli dimensions used and the number of stimuli dimensions learned with a high level of accuracy.

## EXPERIMENT 4

The stimuli dimensions used in our experiments are spatially separable, meaning distinct stimuli dimensions do not overlap on the screen. Additionally, each stimulus in our experiment is presented until participants make a response. It has been shown that the number of eye fixations is greater for multidimensional classification tasks involving stimuli with spatially separable dimensions (Milton & Wills, 2009). Since eye saccades and fixations take time, this could lead to longer response times for participants who use multiple stimuli dimensions for their responses. In Experiment 4, we aimed to test the hypothesis that greater response times for a multidimensional task are solely due to the time taken for eye fixations and movements during the process of gathering information from multiple stimuli features. We controlled for the number of stimuli dimensions required to perform the multidimensional task.

**Figure 2.**
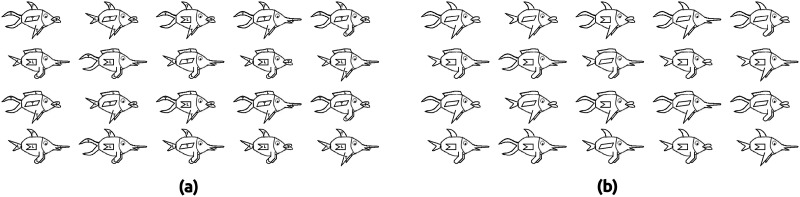
Two sets of stimuli used in Experiment 4. Both sets of stimuli have the same logical structure (see Table 4). (a) Stimuli used in conditions Odd-Even-T and UNI-T. Every feature has either the letter T or the letter L in it. (b) Stimuli used in conditions Odd-Even-A, FR, and UNI-A. Every feature occurs more commonly in either category A or category B (see the logical structure in Table 4).

To test the above hypothesis, we used two types of stimuli in this experiment. One type of stimuli had features containing either the letter T or the letter L (see Figure 2). We devised three multidimensional tasks using different types of stimuli. In the first task (Odd-Even-T), participants needed to check whether the total number of letter T’s in a stimulus was odd or even. This task does not require participants to recall category-relevant information from memory. In the second task (Odd-Even-A), participants needed to check whether the total number of category A features was odd or even. This task is similar to the previous one but requires participants to recall category-relevant information from memory to make the correct response.

If the longer time taken in a multidimensional task is solely due to eye fixations and saccades, then we would expect the mean response time to be the same across the two multidimensional tasks. On the other hand, if the longer time taken in a multidimensional task is also due to the time taken to recall information learned during the training phase, then we would expect the mean response time in the Odd-Even-A condition to be greater than the mean response time in the Odd-Even-T condition.

We also had a third multidimensional task (FR), where participants were trained to categorize a stimulus by examining all its features (family resemblance categorization). We expected the mean response time for both the Odd-Even-A task and the FR task to be greater than the mean response time for the Odd-Even-T task. This would indicate that the longer time taken in a multidimensional task cannot be solely attributed to eye saccades; additional time is needed to recall the information learned during the training phase of the experiment.

Experiment 4 also had two unidimensional task conditions: Uni-T and Uni-A. In the Uni-T condition, participants had to determine whether the top-fin feature contained the letter T. In the Uni-A condition, participants had to determine whether the top-fin feature occurred more commonly in category A.

Differentiation theory posits that humans have access to a preattentive, early stage, holistic comparator which can perform similarity judgements faster compared to unidimensional categorization (Smith & Kemler Nelson, 1984). If participants use the posited first-impression-based, holistic comparator to make similarity judgements in the FR condition, then the mean response time in the FR condition would be less compared to the two unidimensional tasks. We expected the mean response time in FR condition to be greater than the mean response time of unidimensional tasks, which would be consistent with the Combination theory.

### Methods

#### Subjects.

Two hundred and seventy-two participants (35 females; mean age = 19.6 years) participated in this experiment. The participants were undergraduate students at our institution and were randomly assigned to one of the five experimental conditions. We are not aware of any study that compares a multidimensional task requiring recall of information learned during the experiment with another multidimensional task that does not require new information learned during the experiment. We assumed the effect size between Odd-Even-T and Odd-Even-A conditions to be medium. Power analysis with a Cohen’s *f* = .25 (considered a medium effect size), alpha = 0.05, and power = 0.9, indicated a sample size of 250 with 50 participants per condition.

#### Materials.

Figure 2 shows the two sets of stimuli used in the current experiment. The stimuli in Figure 2a were created such that the letters are much smaller than the features and can fit inside them. We aimed to show that participants need additional time to recall category-relevant information learned during the training phase of the experiment. To make this effect harder to show (and to ensure replicability), we made the letters much smaller than the features. Grice et al. (1983) showed that when eye saccades are involved, response time is shorter for a larger target compared to a smaller target. We want to demonstrate that despite the letters being small, it is more effortful to identify the category of each feature compared to identifying the letter inside a feature.

The two sets of stimuli shown in Figure 2 have the same logical structure as shown in Table 4. In Figure 2a, stimuli features corresponding to feature value 1 contain the letter T, and those corresponding to feature value 0 contain the letter L. In Figure 2b, stimuli features corresponding to feature value 1 occur more commonly in category A, and those corresponding to feature value 0 occur more commonly in category B. The stimuli sets are available publicly at OSF: https://osf.io/fkqcy/.

**Table 4.** Logical structure of the two sets of stimuli shown in Figure 2. Stimuli features corresponding to feature value 1 contain the letter T in Figure 2a. Stimuli features corresponding to feature value 1 occur more commonly in category A in Figure 2b.

	Column 1	Column 2	Column 3	Column 4	Column 5
Row 1	11111	10111	11011	11101	11110
Row 2	00000	01000	00100	00010	00001
Row 3	01111	00111	01011	01101	01110
Row 4	10000	11000	10100	10010	10001

#### Procedure.

We used the FindingFive platform to design and run the current experiment (FindingFive, 2024). The experiment had five conditions. In each of the five experimental conditions, participants first learned the task-relevant features (feature training phase). After the feature training phase, participants were trained on the task corresponding to their experimental condition (task training phase). Following task training, participants performed the task again (task test phase). In the final phase of the experiment, participants were asked to report the number of features they used while performing the task. In three of the experimental conditions (Odd-Even-T, Odd-Even-A, and FR), participants were trained on a multidimensional task. In the remaining two conditions (Uni-T and Uni-A), participants were trained to perform a unidimensional task. The stimulus was presented on the screen until participants made a response in each trial across all five conditions of the experiment.

The feature training phase and task training phase of each of the five conditions are explained below.

##### Odd-Even-T.

**Figure 3.**
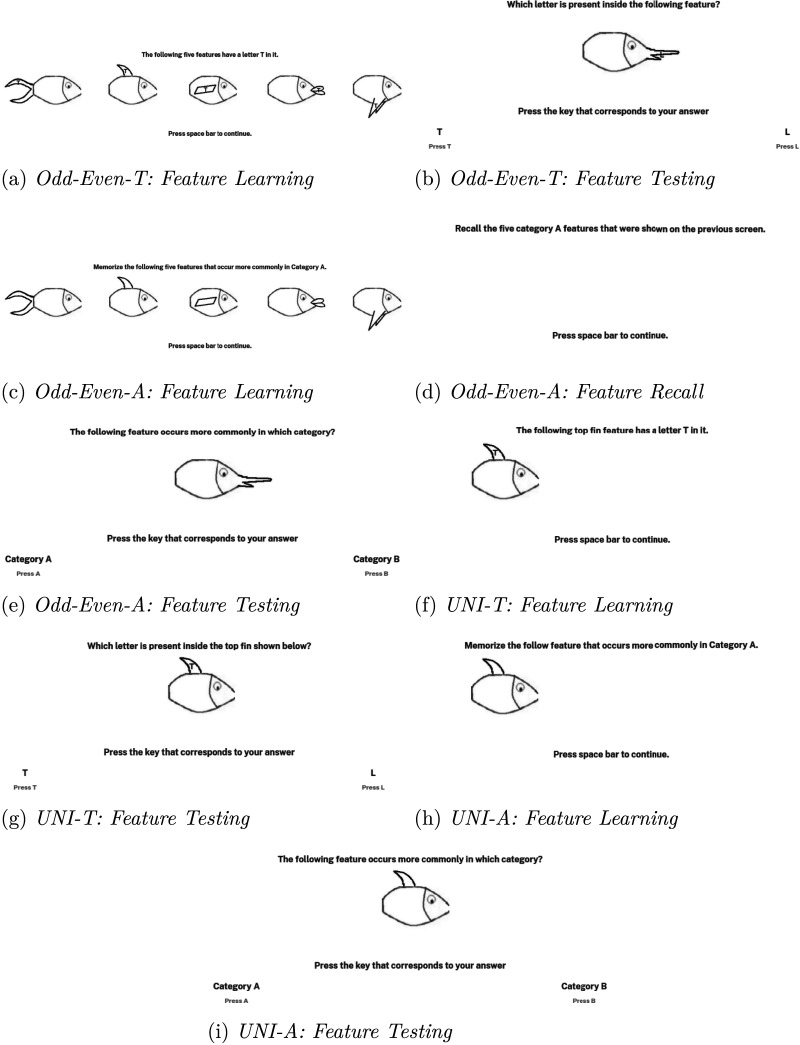
Trial screens from the feature training phase of the experimental conditions. In conditions Odd-Even-T, UNI-T, and UNI-A, participants were familiarized with the task-relevant features. In conditions Odd-Even-A and FR, participants had to memorize the commonly occurring features of categories A and B. The feature training phase of condition FR was identical to condition Odd-Even-A and is not shown above.

Condition Odd-Even-T involved a multidimensional task using stimuli that had either the letter T or L in its features (as shown in Figure 2a). In the feature training phase of this condition, participants were first shown the features containing the letter T (see Figure 3a). Similarly, participants were also familiarized with the features containing the letter L. After feature familiarization, participants were asked to identify the letter inside each of the ten features (see Figure 3b). The ten features were presented in a random order. (The stimuli we used have five dimensions, so there were 10 features in total.) Feedback was given after each feature testing trial to inform participants whether their response was correct. Each feature training block consisted of two feature familiarization trials followed by ten feature testing trials. There were three feature training blocks in the feature training phase of the experimental conditions.

**Figure 4.**
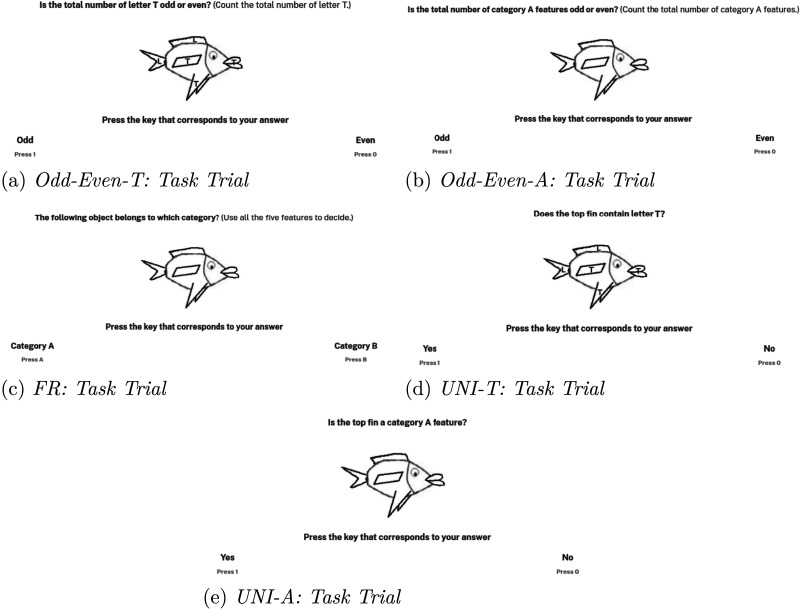
Trial screens from the task phase of the experimental conditions. In condition Odd-Even-T, the task was multidimensional but did not involve memorization of common features of categories. In conditions Odd-Even-A and FR, the task was multidimensional and involved memorization of common features of the categories. In conditions UNI-T and UNI-A, the task was unidimensional.

After the feature training phase, participants proceeded to the task training phase. The task training phase consisted of just one block in which all 20 stimuli shown in Figure 2a were presented in a random order. In each trial, participants had to count the total number of letter T in the presented stimulus (see Figure 4a). Participants had to respond whether the total number was odd or even. This task was designed to ensure that participants would have to look at all the features to make a correct response. Feedback was given to inform participants whether their response was correct. The purpose of the task training phase was to familiarize the participants with the multidimensional task.

##### Odd-Even-A.

Condition Odd-Even-A also involved a multidimensional task similar to condition Odd-Even-T. The main difference was that the stimuli used in Odd-Even-A did not contain any letters in their features (see Figure 2b); participants had to memorize the commonly occurring features of category A and category B. In the feature training phase of the Odd-Even-A condition, participants were first shown the five features that occur commonly in category A (see Figure 3c). Participants were then asked to recall the five category A features shown in the previous screen (see Figure 3d). Similarly, participants were shown the five commonly occurring features of category B and were then asked to recall those features. After memorizing the commonly occurring features of both categories, participants were asked to identify the category in which a given feature occurs more commonly (see Figure 3e). For feature testing, all ten features were presented serially and in a random order. Feedback was given after each feature testing trial to inform participants whether their response was correct. Each feature training block consisted of feature learning and recall trials followed by ten feature testing trials. There were three feature training blocks in the feature training phase of the experiment.

After the feature training phase, participants proceeded to the task training phase. In each trial of the task training phase, participants had to respond whether the total number of category A features in the stimulus was odd or even (see Figure 4b). Just like in the Odd-Even-T condition, feedback was given to inform participants whether their response was correct. The 20 stimuli shown in Figure 2b were presented in a random order.

##### FR.

Condition FR also involved a multidimensional task (family resemblance categorization) using stimuli that did not contain any letters in their features (shown in Figure 2b). Just like condition Odd-Even-A, participants had to memorize the commonly occurring features of category A and category B. The feature training phase of the FR condition was identical to the Odd-Even-A condition.

After the feature training phase, participants proceeded to the task training phase. The FR task asked participants to categorize each stimulus using all five features (see Figure 4c). Participants had to categorize each of the 20 stimuli into either category A or B. The stimuli were presented serially and in a random order. The correct response was category A if three or more features of the stimulus occurred more commonly in category A; otherwise, the correct response was category B. In all the five experimental conditions, participants received feedback for their responses according to the task they were being trained on.

##### UNI-T.

Condition UNI-T involved a unidimensional task using stimuli that had either the letter T or L in its features (as shown in Figure 2a). The unidimensional task involved identifying the letter inside the top-fin feature of the presented stimulus. We selected the top-fin dimension for the unidimensional tasks in conditions UNI-T and UNI-A because it had median visual salience according to a survey we conducted with 56 adult participants. These participants were different from the participants in the four experiments reported in this study. They were shown the 5 stimuli belonging to each of the two categories A and B. The ten stimuli were the training stimuli used in Experiments 1 and 3 but were black in color. For each stimuli dimension, participants were asked to rate its importance or visual salience on a scale of 1 to 7. The survey was conducted using a Google form. The mean ratings (out of 7) obtained were as follows: mouth (5.66), tail (5.02), top-fin (4.75), body-shape (4.5), and bottom-fin (4.39). The top-fin stimuli dimension was found to have median importance or visual salience.

In the feature training phase of condition UNI-T, participants were first shown the task-relevant feature containing the letter T (see Figure 3f). Similarly, participants were shown the task-relevant feature containing the letter L. After feature familiarization, participants were asked to identify the letter inside each of the two features that formed the top-fin stimuli dimension (see Figure 3g). The two task-relevant (top-fin) features were presented in a random order. Feedback was given after each feature testing trial. Each feature training block consisted of two feature familiarization trials followed by two feature testing trials. There were three feature training blocks in the feature training phase of the experiment.

After the feature training phase, participants proceeded to the task training phase. The task training phase consisted of just one block in which all 20 stimuli shown in Figure 2a were presented in a random order. In each trial, participants had to tell whether the top-fin feature contained the letter T or the letter L (see Figure 4d). Feedback was given to inform participants whether their response was correct.

##### UNI-A.

Condition UNI-A involved a unidimensional task using stimuli that did not contain any letters in their features (shown in Figure 2b). In the unidimensional task of condition UNI-A, participants had to tell whether the top-fin feature occurred more commonly in category A or in category B. In the feature training phase of this condition, participants were first shown the task-relevant feature that occurred more commonly in category A (see Figure 3h). Similarly, participants were shown the task-relevant feature that occurred more commonly in category B. After feature familiarization, participants were asked to identify the category in which the top-fin feature occurred more commonly (see Figure 3i). The two task-relevant (top-fin) features were presented in a random order. Feedback was given after each feature testing trial. Each feature training block consisted of two feature familiarization trials followed by two feature testing trials. There were three feature training blocks in the feature training phase of the experiment.

After the feature training phase, participants proceeded to the task training phase. The task training phase consisted of just one block in which all 20 stimuli shown in Figure 2b were presented in a random order. In each trial, participants had to tell whether the top-fin feature occurred more commonly in category A or in category B (see Figure 4e). Feedback was given to inform participants whether their response was correct.

In each of the five experimental conditions, the task training phase was followed by the task test phase. The task test phase was identical to the task training phase of the corresponding experimental condition. Feedback regarding the correctness of responses was provided after each response to ensure that participants tried to be as accurate as possible and did not change their response strategy during the task. After the task test phase, participants were asked to report the number of stimuli dimensions they used to perform the task. They could choose an integer between 0 and 5 (both inclusive).

### Results and Discussion

**Figure 5.**
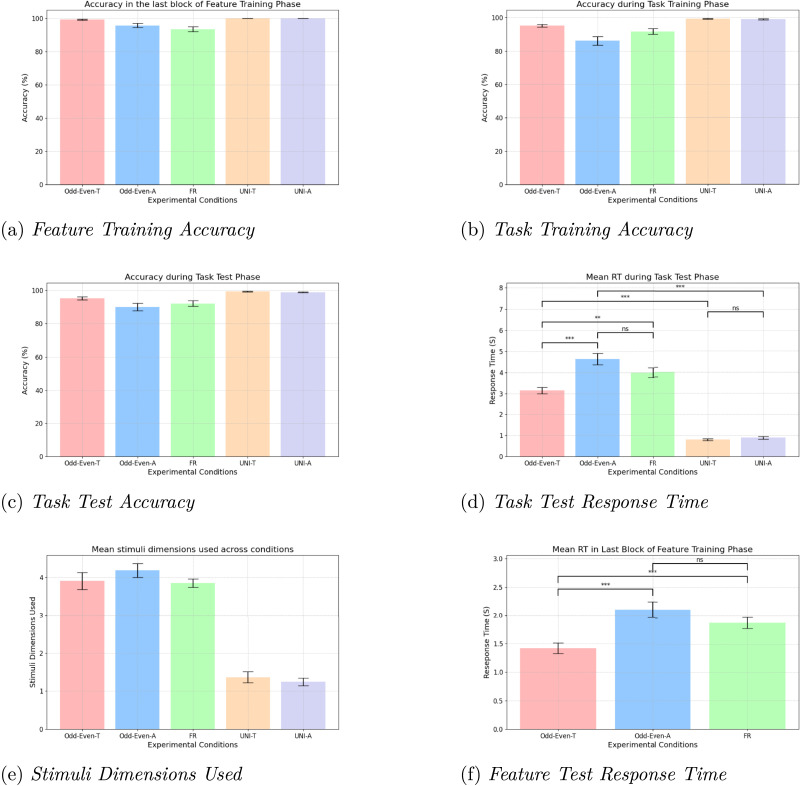
Results for the experimental conditions in Experiment 4. (a) Accuracy in the final block of the feature training phase. (b) Accuracy in the task training phase. (c) Accuracy in the task test phase. (d) Response time in the task test phase. (e) Stimuli dimensions used for the task. (f) Response time in the final block of the feature training phase for multidimensional tasks.

The number of participants in conditions Odd-Even-T, Odd-Even-A, FR, UNI-T, and UNI-A were 55, 54, 54, 55 and 54 respectively. We did not omit any participant’s data from our analysis. We first report the results of the feature training phase in Figure 5a. The accuracy (in percentage) for the feature test trials in the last (third) block of the feature training phase were as follows: Odd-Even-T (*M* = 99.27, *SD* = 2.60), Odd-Even-A (*M* = 95.74, *SD* = 8.94), FR (*M* = 93.52, *SD* = 10.74), UNI-T (*M* = 100, *SD* = 0), and UNI-A (*M* = 100, *SD* = 0). This shows that at the end of the feature training phase, participants learned the task-relevant features with a high level of accuracy.

Figure 5b shows the accuracy for the task trials in the task training phase. The accuracy (in percentage) for the five conditions were as follows: Odd-Even-T (*M* = 95.27, *SD* = 5.83), Odd-Even-A (*M* = 86.20, *SD* = 18.81), FR (*M* = 91.76, *SD* = 12.66), UNI-T (*M* = 99.36, *SD* = 1.92), and UNI-A (*M* = 99.07, *SD* = 2.17). This shows that at the end of the task training phase, participants learned the task corresponding to each condition with a high level of accuracy. The accuracy in conditions UNI-T and UNI-A exhibited a ceiling effect, suggesting that unidimensional tasks were easier to learn compared to the multidimensional tasks.

Figure 5c shows the accuracy for the task trials in the task test phase. The accuracy (in percentage) for the five conditions were as follows: Odd-Even-T (*M* = 95.18, *SD* = 7.07), Odd-Even-A (*M* = 90.0, *SD* = 16.19), FR (*M* = 92.13, *SD* = 12.23), UNI-T (*M* = 99.27, *SD* = 1.76), and UNI-A (*M* = 98.80, *SD* = 2.14). This shows that participants performed the unidimensional and the multidimensional tasks with a high level of accuracy across the experimental conditions. Once again, we see a ceiling effect for the unidimensional tasks.

Figure 5d shows the mean response time (in seconds) for the task trials in the task test phase, which were as follows: Odd-Even-T (*M* = 3.15, *SD* = 1.12), Odd-Even-A (*M* = 4.64, *SD* = 1.92), FR (*M* = 4.00, *SD* = 1.65), UNI-T (*M* = .80, *SD* = .32), and UNI-A (*M* = .89, *SD* = .46). We performed a one-way between-subjects ANOVA, with experimental task conditions as the factor, on the mean response time. The ANOVA results showed a significant effect of experimental task conditions on the mean response time, *F*(4, 267) = 105.22, *p* < .0001, *η*^2^ = .61. Post-hoc analyses were performed using Tukey’s HSD test (two-tailed), and the results are shown in Table 5.

**Table 5.** Tukey HSD test results for response time (s) in the task test phase across the five experimental conditions.

Group 1	Group 2	Mean Diff (s)	Adjusted *p*-value	Reject Null
Odd-Even-T	Odd-Even-A	1.49	.001	True
Odd-Even-T	FR	.85	.006	True
Odd-Even-T	UNI-T	−2.35	.001	True
Odd-Even-T	UNI-A	−2.26	.001	True
Odd-Even-A	FR	−.65	.067	False
Odd-Even-A	UNI-T	−3.84	.001	True
Odd-Even-A	UNI-A	−3.75	.001	True
FR	UNI-T	−3.20	.001	True
FR	UNI-A	−3.11	.001	True
UNI-T	UNI-A	.09	.90	False

Post-hoc analyses using Tukey’s HSD test showed that the response time for the Odd-Even-A task (*M* = 4.64, *SD* = 1.92) was significantly greater than the response time for the Odd-Even-T task (*M* = 3.15, *SD* = 1.12); *t*(107) = 4.91, *p* < .0001, *d* = 0.94. The time taken to count category A features in the stimuli was significantly greater than the time taken to count the number of letter T’s, despite the letters being much smaller compared to the stimuli features. This indicates that the additional time required for a multidimensional task is not solely due to eye fixations and saccades; participants need more time to recall the feature-category associations learned during the experiment.

Post-hoc analyses using Tukey’s HSD test also showed that the response time for the FR task (*M* = 4.00, *SD* = 1.65) was significantly greater than the response time for the Odd-Even-T task; *t*(107) = 3.10, *p* = .002, *d* = .59. The Odd-Even-T task involves processing each dimension individually to check for the presence of the letter “T” and then computing whether the total count is odd or even. This task does not rely on similarity-based processing. In contrast, the FR task is similarity-based and more closely resembles natural category membership decisions, where members share common features. Evolution may have equipped humans with a primitive, direct mechanism for similarity recognition—a pre-attentive, first-impression-based, low-resource processing mode (Kemler Nelson, 1984; Smith & Kemler Nelson, 1984; Smith & Shapiro, 1989). If participants can engage this low-resource, holistic processor for similarity judgments, the time required for the FR task should be notably shorter than for the Odd-Even-T task, which requires processing individual dimensions before computing the correct response. Our results, however, suggest that participants did not use this fast, pre-attentive, holistic processor for the overall similarity-based categorization in the FR condition.

Differentiation theory proposes that humans have access to a pre-attentive, early-stage, holistic comparator that allows faster similarity judgments compared to unidimensional categorization (Smith & Kemler Nelson, 1984). In the FR condition, participants had the opportunity to engage this early-stage, holistic processing mode, which is thought to be faster. In contrast, the UNI conditions required a more analytic mode of categorization, involving later-stage processing. Post-hoc analyses using Tukey’s HSD test showed that the response time for the FR task (*M* = 4.00, *SD* = 1.65) was significantly greater than the response time for the Uni-A task (*M* = .89, *SD* = .46), *t*(106) = 13.22, *p* < .0001, *d* = 2.55; and the Uni-T task (*M* = .80, *SD* = .32), *t*(107) = 13.99, *p* < .0001, *d* = 2.68. This suggests that participants did not employ the less resource-intensive, pre-attentive mechanism for holistic categorization as predicted by Differentiation theory. Table 5 presents the pairwise Tukey’s HSD test results for response times in the task test phase of the experiment.

Figure 5e shows the number of stimuli dimensions that participants reported using after the task test phase. The reported dimensions were as follows: Odd-Even-T (*M* = 3.91, *SD* = 1.64), Odd-Even-A (*M* = 4.19, *SD* = 1.33), FR (*M* = 3.85, *SD* = .83), UNI-T (*M* = 1.36, *SD* = 1.07), and UNI-A (*M* = 1.24, *SD* = .74). These results indicate that participants did not switch to a different strategy as the experiment progressed. Participants in the Odd-Even-T, Odd-Even-A, and FR conditions consistently used a multidimensional strategy, while participants in the UNI-T and UNI-A conditions used a unidimensional strategy.

In the feature training phase of conditions Odd-Even-A and FR, participants learned the category associated with each feature. In condition Odd-Even-T, participants learned to identify the letter inside each feature. We wanted to check whether participants need more time to recall the category associated with each feature compared to identifying the letter inside a feature. We analyzed the response times in the last block of the feature training phase for conditions Odd-Even-T, Odd-Even-A, and FR. We excluded the unidimensional conditions because participants in those conditions were not trained on all the ten stimuli features (only two features were task-relevant). Figure 5f shows that the response time in the last block of the feature training phase were as follows: Odd-Even-T (*M* = 1.42, *SD* = .68), Odd-Even-A (*M* = 2.10, *SD* = 1.03), and FR (*M* = 1.88, *SD* = .72). The ANOVA results showed a significant effect of the feature training task on the mean response time, *F*(2, 160) = 9.67, *p* < .001, *η*^2^ = .11. Tukey’s HSD test indicated that the mean response time in the last block of the feature learning phase for condition Odd-Even-T was significantly less compared to the mean response time for both condition Odd-Even-A (*t*(107) = 4.08, *p* < .001, *d* = 0.78) and condition FR (*t*(107) = 3.39, *p* = 0.001, *d* = 0.65). Tukey’s HSD test indicated that the mean response time for condition Odd-Even-A was not significantly different from the mean response time for condition FR (*t*(106) = 1.32, *p* = 0.1896, *d* = 0.25). (This was expected because the feature learning phase for conditions Odd-Even-A and FR were identical.) These results further show that participants need significantly more time to recall the information learned during the experiment compared to identifying a letter inside a stimuli feature, even when the letters are much smaller compared to the stimuli features.

Our results indicate that participants do not use the fast, early-stage holistic comparator for making similarity-based categorizations in the FR task. Additionally, the longer time required for multidimensional tasks, such as the Odd-Even-A and FR tasks, is not solely due to eye movements. Overall, our findings suggest that holistic processing (involving all features) of stimuli is more effortful and time-consuming compared to unidimensional categorization.

## GENERAL DISCUSSION

The current study uses stimuli with spatially separable features. By spatially separable, we mean stimuli dimensions that do not overlap. Kemler Nelson (1984) and Smith and Shapiro (1989) also used stimuli with spatially separable dimensions. Kemler Nelson (1984) showed a greater preference for overall similarity-based categorization under incidental (observation) learning compared to intentional (classification) learning. Smith and Shapiro (1989) demonstrated a greater preference for overall similarity-based categorization under load condition compared to no load condition, indicating that overall similarity-based categorization is faster and less effortful. Wills et al. (2015) argued that classical analysis does not differentiate between overall similarity-based grouping and non-criterial unidimensional grouping. Instead of classical analysis, Wills et al. (2015) used response set analysis to show that very few participants performed overall similarity-based categorization, which is slower and more effortful, as predicted by Combination theory.

In the current study, we employed both incidental (observation) learning and intentional (classification) learning in Experiments 1 and 2, and only intentional learning in Experiment 3. Our results show that overall similarity-based categorization is preferred by fewer participants. We used participants’ categorization descriptions to determine the number of stimuli dimensions used for categorization. Our results consistently show that using more dimensions is more effortful and takes more time, which is consistent with the predictions of Combination theory.

To perform multidimensional categorization using stimuli with spatially separable features, participants need multiple eye fixations and saccades (Milton & Wills, 2009). So, the additional time required to classify using more dimensions could be due to the additional eye fixations and saccades needed to gather information about multiple features. In Experiment 4, we tested the hypothesis that the increased time for multidimensional categorization can be explained solely by the eye fixations and saccades required to gather the necessary information. Our results show that participants need additional time to recall the feature-category associations, which makes the multidimensional task slower and more effortful.

Our results do not address whether Combination theory would be supported when visual stimuli with spatially non-separable dimensions are used. Smith and Kemler Nelson (1984) used stimuli with two dimensions—size and brightness. These dimensions are not spatially separable, allowing information from both dimensions to be gathered without multiple eye fixations and saccades. Ward (1983) also used stimuli with overlapping dimensions—length of a line and density of dots forming the line. Both Smith and Kemler Nelson (1984) and Ward (1983) reported that rapid responses were associated with an increase in overall similarity-based grouping, which supports Differentiation theory. Wills et al. (2015) attempted to replicate these results and found that they could be explained by both Combination theory and Differentiation theory. As mentioned earlier, this is because classical analysis does not differentiate between overall similarity-based grouping and non-criterial unidimensional grouping. Further studies are needed to determine whether Combination theory would apply to visual stimuli with features that are spatially non-separable.

Obasih et al. (2023) studied the effect of different training regimes on complex, multidimensional auditory stimuli and showed that learning is equivalently robust for auditory stimuli across different training regimes. Roark and Holt (2019) investigated whether acoustic dimensions are perceptually integral and difficult to attend to selectively. The results showed a bias towards integrating across the acoustic dimensions rather than selectively attending to one of them. Further studies are needed to test the predictions of Combination and Differentiation theories on auditory category learning involving stimuli with auditory dimensions that are separable as well as non-separable in time.

In the Supplemental material, we used Bayesian modeling to show that a stimuli dimension is used for categorization only when it is learned with a high level of accuracy. We compared different Bayesian models in our analysis. Our results show that the model that allows a partially diagnostic dimension to be used for categorization only when it is learned with high accuracy provides a better explanation for the data across the first three experiments. In the first three experiments, the results of linear regression analysis show that participants who used multiple stimuli dimensions also learned multiple dimensions with 100% accuracy. Our results are consistent with the finding that pre-training participants to learn the family resemblance structure of the categories (with high accuracy) leads to overall similarity-based categorization (Thomas & Srinivasan, 2023; Wills et al., 2020). These studies also report an increase in mean response time when participants perform multidimensional categorization, which is consistent with Combination theory.

Combination theory only makes predictions about how a multidimensional strategy is applied. It does not predict how a multidimensional strategy is learned. Seitz et al. (2023) showed that—when there is no criterial attribute—classification learners need many trials to achieve a slightly difficult learning criterion. (The learning criterion used by Seitz et al. (2023) was 80% accuracy in the previous 100 trials and 100% accuracy in the previous 24 trials.) Additionally, time pressure made participants give more random responses instead of overall similarity-based responses (Seitz et al., 2023). These results show that learning a multidimensional strategy is slower and more effortful. Further studies are needed to establish the connection between Combination theory and how people learn a multidimensional task.

## CONCLUSION

In this study, we used participants’ categorization descriptions to compare two theories, Combination and Differentiation, which make different predictions about the order in which information about a stimulus is processed during categorization. Our results show that participants who used more stimuli dimensions took more time to categorize a stimulus. Additionally, the number of stimuli dimensions used is positively correlated with the number of stimuli dimensions learned with high accuracy. Our findings also indicate that the additional time taken in an overall similarity-based categorization task is not solely due to eye movements; Participants need more time to recall the feature-category associations learned during the experiment, which makes holistic processing (involving all features) slower and more effortful.

Overall, our results support the Combination theory but not the Differentiation theory. We used visual stimuli with spatially separable dimensions. Further studies are needed to determine whether Combination theory applies to stimuli with spatially non-separable dimensions.

## ACKNOWLEDGMENTS

We acknowledge the use of ChatGPT, a large language model developed by OpenAI, for assistance in correcting grammatical errors, typos, and some phrases in this manuscript (OpenAI, 2024). The tool was used to make minor changes to improve the readability and clarity of the text.

## FUNDING INFORMATION

This research received no specific grant from any funding agency, commercial, or not-for-profit sectors.

## AUTHOR CONTRIBUTIONS

Sujith Thomas: Conceptualization; Data curation; Formal analysis; Methodology; Writing – original draft; Writing – review & editing. Aditya Kapoor: Data curation; Formal analysis; Methodology. Narayanan Srinivasan: Conceptualization; Supervision; Writing – review & editing.

## DATA AVAILABILITY STATEMENT

The behavioural data, stimuli and analyses code are available publicly at OSF: https://osf.io/fkqcy/.

## Notes

^1^ The results of linear regression indicated that the mean response time in the transfer phase is a significant predictor of the number of stimuli dimensions used, *β* = .60, *t*(37) = 4.57, *p* < .001, *R*^2^ = .36, adjusted *R*^2^ = .34. The results of linear regression indicated that the accuracy in the all features test phase is a significant predictor of the number of stimuli dimensions used, *β* = .39, *t*(37) = 2.60, *p* = .01, *R*^2^ = .16, adjusted *R*^2^ = .13. The results of linear regression indicated that the number of stimuli dimensions learned with 100% accuracy is a significant predictor of the number of stimuli dimensions used, *β* = .47, *t*(37) = 3.22, *p* = .003, *R*^2^ = .22, adjusted *R*^2^ = .20.^2^ The results of linear regression indicated that the mean response time in the transfer phase is a significant predictor of the number of stimuli dimensions used, *β* = .63, *t*(34) = 4.74, *p* < .001, *R*^2^ = .40, adjusted *R*^2^ = .38. Linear regression analysis showed that the number of stimuli dimensions learned with 100% accuracy is a significant predictor of the number of stimuli dimensions used, *β* = .46, *t*(34) = 2.99, *p* = .005, *R*^2^ = .21, adjusted *R*^2^ = .19. Linear regression analysis showed that the accuracy in the all features test phase is a significant predictor of the number of stimuli dimensions used, *β* = .39, *t*(34) = 2.48, *p* = .018, *R*^2^ = .15, adjusted *R*^2^ = .13.^3^ The results of linear regression indicated that the mean response time in the transfer phase is a significant predictor of the number of stimuli dimensions used, *β* = .48, *t*(44) = 3.67, *p* < .001, *R*^2^ = .23, adjusted *R*^2^ = .22. The results of linear regression indicated that the number of stimuli dimensions learned with 100% accuracy is a significant predictor of the number of stimuli dimensions used, *β* = .41, *t*(44) = 2.94, *p* = .005, *R*^2^ = .17, adjusted *R*^2^ = .15. The results of linear regression indicated that the accuracy in the all features test phase is not a significant predictor of the number of stimuli dimensions used, *β* = .14, *t*(44) = .94, *ns*, *R*^2^ = .02, adjusted *R*^2^ = .00.

## Supplementary Material


