# Supplementary material for: Combination and Differentiation Theories of Categorization: A Comparison Using Participants’ Categorization Descriptions
Source: Open Mind (Camb). 2025 Feb 8;9:266–89. doi: 10.1162/opmi_a_00187 (PMC11850022; doi:10.1162/opmi_a_00187)
Supplement: Supplementary file 1 [file opmi-09-266-s001.pdf]

Combination and Differentiation theories of categorization : A comparison using participants' categorization descriptions (Supplemental Document)

Bayesian Modelling

Exploratory data analysis in the main article revealed that there is a significant correlation between how well participants learned the diagnosticity of stimuli dimensions and the use of a multidimensional categorization strategy. We wanted to provide further evidence for this using Bayesian modelling analysis. Our goal was to show that there is a minimum accuracy with which a stimuli dimensions must be learned for it to be used during categorization in the transfer phase. In the rest of this article, we refer to the perfectly diagnostic stimuli dimension as the CA (criterion attribute) dimension and the partially diagnostic dimensions as the FR (family resemblance) dimensions.

In our models, we have used a distance function that finds the distance of a transfer stimulus from the prototype of a category. The distance function is similar to the distance functions that has been used for the prototype model and the generalized context model (Minda & Smith, 2011; Nosofsky, 2011). Though we use distances from the category prototypes, our goal is not to argue in favour of the prototype model. The goal of our Bayesian modelling is to take into account how well a stimuli dimension is learned while predicting the categorization behaviour.

Figure 1 shows the Bayesian model that we have used. The shaded nodes represent the observed variables. The unshaded nodes having a single border represent the free parameters whose value depends on a prior probability distribution. The square-shaped nodes take discrete values, while circular nodes take continuous values. The nodes with double borders are the deterministic nodes whose value depend on the parent node(s).

We have used three observed variables: $\vec{x_i}$, $\vec{t_k}$ and r^A_{ki}. The variable $\vec{x_i}$ is a five dimensional vector that corresponds to the *logical* representation of the ith transfer stimulus. As described earlier, we have used five different sets of stimuli, where each set had a different stimuli dimension forming the CA dimension. We have used a logical representation in which the first dimension is always the CA dimension. Suppose that for a stimuli set the third stimuli dimension is the CA dimension. Then the third stimuli dimension is mapped to the first logical dimension, the fourth stimuli dimension is mapped to the second logical dimension and so on. After the fifth stimuli dimension we rotate back to the first stimuli dimension and continue the mapping.

The second observed variable that we have used is $\vec{t_k}$, which is also a five dimensional vector. The jth component of vector $\vec{t_k}$ (denoted by t_{kj}) contains the accuracy of the kth participant for the jth *logical* dimension. This means that the first component of vector $\vec{t_k}$ will always contain the accuracy for the CA dimension. In each block of the all features test phase, every stimuli dimension was tested twice (because each dimension has two features). In total there were three blocks. This means that each stimuli dimension was tested six times in the all features test phase. The accuracy

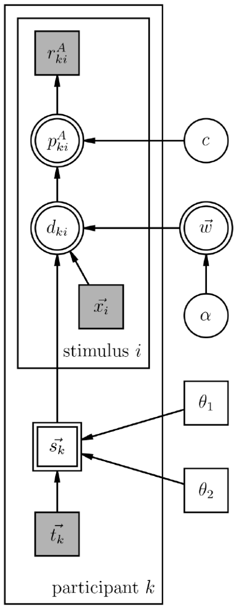

$$c \sim Uniform(0, 20)$$
$$\theta_1 \sim DUniform([-.01, .16, .32, .49, .66, .82, .99])$$
$$\theta_2 \sim DUniform([-.01, .16, .32, .49, .66, .82, .99])$$
$$s_{kj} \leftarrow \begin{cases} Step(t_{kj} - \theta_1), & \text{if } j = 1 \\ Step(t_{kj} - \theta_2), & \text{if } j \neq 1 \end{cases}$$
$$\alpha \sim Uniform(0, 1)$$
$$w_j \leftarrow \begin{cases} 1 - \alpha, & \text{if } j = 1 \\ \alpha/4, & \text{if } 2 \leq j \leq 5 \end{cases}$$
$$d^a_{ki} \leftarrow \sum_{j=1}^{5} w_j s_{kj} |x_{ij} - a_j|$$
$$d^b_{ki} \leftarrow \sum_{j=1}^{5} w_j s_{kj} |x_{ij} - b_j|$$
$$p^A_{ki} \leftarrow 1 - \frac{d^a_{ki} + c}{d^a_{ki} + d^b_{ki} + 2c}$$
$$r^A_{ki} \sim Binomial(p^A_{ki}, 3)$$

Figure 1
Graphical representation describes the stochastic processes that generate the observed data from unobserved parameters for model I.

for the jth *logical* dimension for the kth participant (i.e. t_{kj}) was found by dividing the number of correct responses for each dimension by 6. So, t_{kj} can have one of the following discrete values: 0, .17, .33, .5, .67, .83 or 1. The accuracy values have been rounded to two decimal places.

The third observed variable r^A_{ki} tells us how many times the kth participant categorized the ith transfer stimulus to category A. In the transfer phase, there were three blocks. So, every participant categorized each transfer stimulus three times. For this reason, the value of variable r^A_{ki} will be 0, 1, 2 or 3. The deterministic parameter p^A_{ki} gives the probability that k^{th} participant will categorize a transfer stimulus $\vec{x_i}$ to category A. In our model, the observed variable r^A_{ki} is generated from a Binomial distribution with probability p^A_{ki} and three Bernoulli trials (see Figure 1).

The probability p^A_{ki} was determined using distances d^a_{ki} and d^b_{ki}. Distance d^a_{ki} is the distance of stimulus $\vec{x_i}$ from the prototype of category A (denoted by $\vec{a}$). The prototype $\vec{a}$ will contain the diagnostic features (both CA and FR) of category A. We have found the distance d^a_{ki} as follows:

$$d^a_{ki} = \sum_{j=1}^{5} w_j s_{kj} |x_{ij} - a_j| \qquad (1)$$

where w_j is the attentional weight for logical dimension j and s_{kj} is a parameter that can be either 0 or 1. The parameters w_j and s_{kj} are explained below. In Eqn. (1), $|x_{ij} - a_j|$ will be 0 if $\vec{x_i}$ and $\vec{a}$ have the same j^{th} feature, otherwise $|x_{ij} - a_j|$ will be 1. So the distance d^a_{ki} will increase if stimulus $\vec{x_i}$ contains fewer diagnostic features of category A. We find the distance d^b_{ki} in a manner similar to Eqn. (1), where $\vec{b}$ denotes the prototype of category B.

In Eqn. 1, parameter s_{kj} determines whether the kth participant used the jth dimension for categorization. We have hypothesized that participant k would use the jth dimension only when the accuracy t_{kj} is above some threshold (θ). To capture this relation we have used a unit $Step()$

function.

$$s_{kj} \leftarrow \begin{cases} Step(t_{kj} - \theta_1), & \text{if } j = 1 \\ Step(t_{kj} - \theta_2), & \text{if } 2 \leq j \leq 5 \end{cases} \tag{2}$$

The value of the $Step()$ function is 1 when its argument is positive, otherwise it is 0. So, parameter s_{kj} will be 1 if the accuracy t_{kj} is above some threshold, otherwise s_{kj} will be 0. If s_{kj} is 0, then the jth logical dimension will be ignored while computing the distance in Eqn. (1). In Eqn (2), we have used threshold θ_1 for the CA dimension (i.e. $j = 1$), and threshold θ_2 for the four FR dimensions (i.e. $2 \leq j \leq 5$). This is because we are working with the logical representation where different stimuli dimensions can map to the same logical dimension depending on the stimuli set being used. For this reason, we did not want to differentiate between the different FR dimensions and have used the same threshold θ_2.

The threshold parameters (θ_1 and θ_2) are generated from a uniform prior distribution over the following discrete values: $-.01$, .16, .32, .49, .66, .82 and .99. These values are .01 less than the seven accuracy values that t_{kj} can take. Our primary objective was to check whether there is a minimum accuracy below which a stimuli dimension is ignored. Since the observed variable t_{kj} takes discrete values, we have assumed that the threshold parameters also take discrete values [1] .

In Eqn. (1), we have used attentional weights w_j for each dimension. These weights are determined as follows:

$$w_j \leftarrow \begin{cases} 1 - \alpha, & \text{if } j = 1 \\ \alpha/4, & \text{if } 2 \leq j \leq 5 \end{cases} \tag{3}$$

where α is a parameter having a uniform prior distribution in the interval $(0, 1)$. Determining the weights in the above manner ensures that all the weights sum to 1. Eqn. (3) allows the weights for the CA dimension to be different from the FR dimensions. However, the attentional weights for the FR dimensions are the same. The reason for this is (again) that we don't want to differentiate between different FR dimensions in the logical representation for the stimuli.

If the distance d^a_{ki} is small, it would mean that stimulus $\vec{x_i}$ has many diagnostic features of category A; therefore, the probability p^A_{ki} must be closer to 1. The probability p^A_{ki} was determined from distances d^a_{ki} and d^b_{ki} as follows:

$$p^A_{ki} = 1 - \frac{d^a_{ki} + c}{d^a_{ki} + d^b_{ki} + 2c} \tag{4}$$

where d^a_{ki} and d^b_{ki} lie in the range 0 to 1. The distances d^a_{ki} and d^b_{ki} can both become zero for a participant who has learned all the dimensions poorly (low accuracy), because s_{kj} will be 0 for all the dimensions. To avoid the divide-by-zero error in Eqn. (4), we have added parameter c and $2c$ in the numerator and the denominator respectively. If both the distances become zero, then the probability p^A_i will become .5 because of c and $2c$ present in the numerator and the denominator respectively. This will model the fact that the participant who is not sure of the diagnosticity of any of the dimensions is probably giving random categorization responses. Parameter c has a uniform prior distribution in the interval $(0, 20)$. If the values of parameter c sampled from the marginal posterior distribution are very small (close to zero), then it would indicate that the

[1]We could have used a continuous prior distribution (i.e $Uniform(0, 1)$) for the threshold parameters. In that case, the posterior distribution would lie in the interval $(\frac{4}{6}, \frac{5}{6})$ and $(\frac{5}{6}, 1)$ instead of being concentrated at .82 and .99 respectively (See Figure 2). Since we are only interested in the minimum accuracy with which a dimension should be learned, we have used discrete values.

Table 1
Mean and standard deviation estimates of marginal posterior distribution for the parameters θ_1, θ_2, w_1 and w_2 for all the three experiments. The data is shown in $Mean(SD)$ format.

Exp.	θ_1	θ_2	w_1	w_2
1	.83(.03)	.99(0)	.58(.02)	.10(.01)
2	.74(.08)	.82(.003)	.44(.02)	.14(.005)
3	.82(0)	.99(.02)	.43(.02)	.14(.005)

distance measures are able to predict the probability p_{ki}^A. On the other hand, if the values of parameter c are very large compared to the distances, then p_{ki}^A will be close to .5. This would indicate that our distance measures are not effective in predicting the probability p_{ki}^A.

Results: Marginal Posterior Distributions

We have used the R2jags library (Plummer et al., 2003; Su & Yajima, 2012) in R to obtain samples from the joint posterior distribution of parameters using Gibbs sampling. We have monitored parameter c, the attentional weights ($\vec{w}$) and the accuracy thresholds (θ_1 and θ_2). All our results are based on three chains of 4,000 samples each (total 12,000 samples). Each of the three chains had a burn-in of 1,000 samples, and the samples were thinned by taking every tenth sample. The convergence of the three chains were checked using the standard $\hat{R}$ statistic (Brooks & Gelman, 1998).

Our model has four free parameters: c, α, θ_1 and θ_2. We discuss the results separately for each of the three experiments. We had 450 data points (45 participants $\times$ 10 transfer stimuli) for Experiment 1, 450 data points (45 participants $\times$ 10 transfer stimuli) for Experiment 2, and 500 data points (50 participants $\times$ 10 transfer stimuli) for Experiment 3. We obtained $12,000$ samples from the posterior distribution for the data in each of the three experiments. The results discussed below are based on the $12,000$ samples obtained for the corresponding experiment.

The mean and the standard deviation of the marginal posterior distribution of parameter c were found to be ($M = .019, SD = .006$), ($M = .0066, SD = .0031$) and ($M = .0087, SD = .0027$) for Experiments 1, 2 and 3 respectively. The distance measures d_{ki}^a and d_{ki}^b lie in the range 0 to 1. The values of parameter c were small compared to the distance measures d_{ki}^a and d_{ki}^b. This shows that distance measures are able to predict the probability p_{ki}^A as given in Eqn. (4).

Figure 2 shows the posterior distribution for the parameters w_1, θ_1, w_2 and θ_2 for all the three experiments. The histograms at the top and the right of the graph indicate the marginal posterior distributions for the threshold parameter and the attentional weight parameter respectively. The bars in the histogram show the number of samples (out of 12,000) that fall under different ranges of the parameter values. Table 1 shows the mean and the standard deviation for marginal posterior distribution of the parameters w_1, θ_1, w_2 and θ_2.

Table 1 shows that the mean for parameter θ_1 is less in Experiment 2. Figure 2c shows that the marginal posterior distribution for θ_1 in Experiment 2 has two modes (at .66 and .82). This is because, in Experiment 2, no participant had an accuracy of .67 for the CA dimension. For this reason, the likelihood function value would remain the same if $\theta_1 = .82$ or $\theta_1 = .66$. Since we are using uniform priors for θ_1 in model I, the posterior probabilities would remain the same for $\theta_1 = .66$ and $\theta_1 = .82$ (because both prior probability and likelihood are the same). For this reason, we have two modes in the histogram and the mean for parameter θ_1 is less for Experiment 2 compared to the other two experiments (see Table 1). Overall, the results show that the mean of the threshold parameters for both the CA dimension (θ_1) and the FR dimensions (θ_2) were high

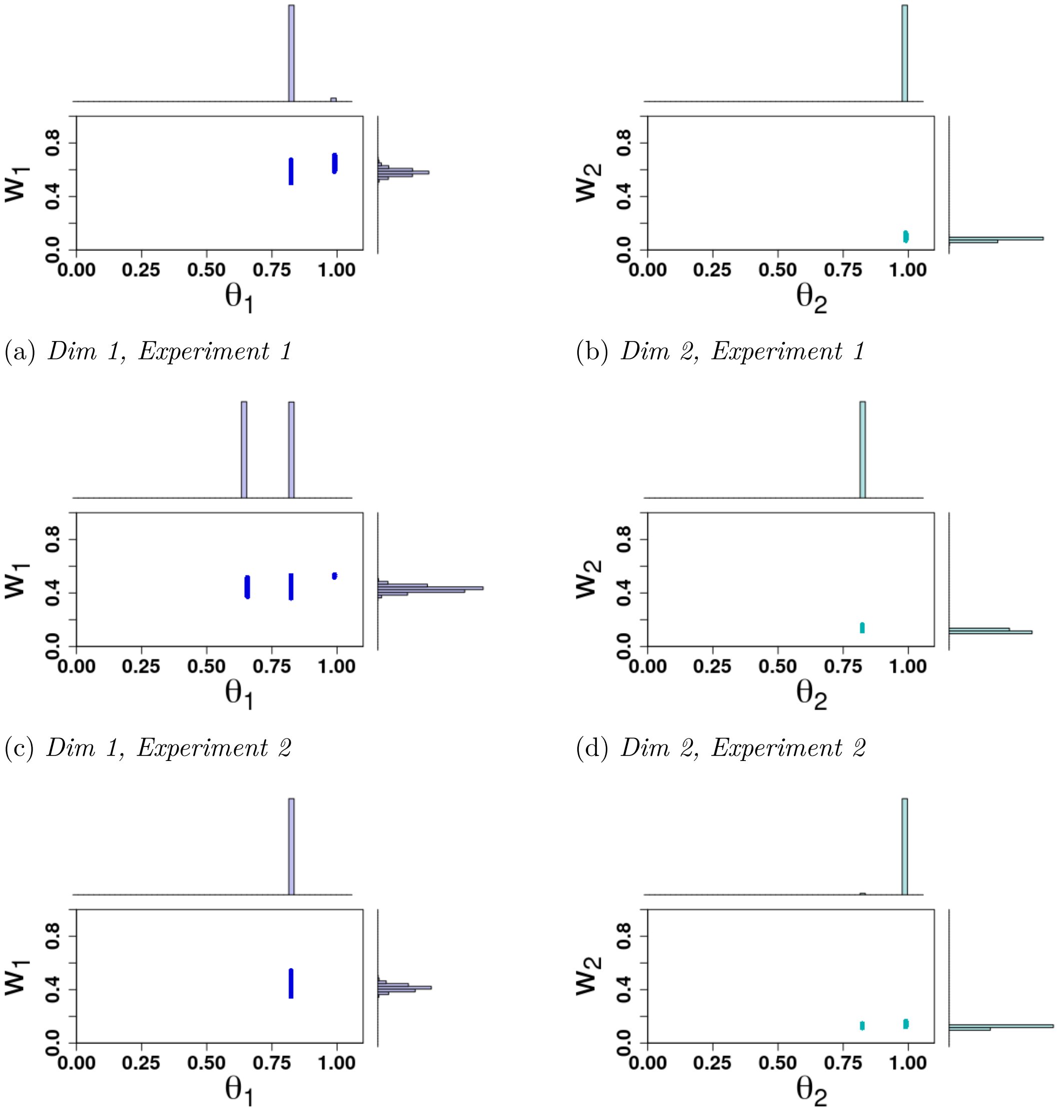

(a) *Dim 1, Experiment 1*
(b) *Dim 2, Experiment 1*
(c) *Dim 1, Experiment 2*
(d) *Dim 2, Experiment 2*
(e) *Dim 1, Experiment 3*
(f) *Dim 2, Experiment 3*

Figure 2

The graphs indicate the joint posterior distribution for attentional weight and threshold parameters corresponding to Model I ((Figure 1)). The caption for each graph shows the logical dimension and the experiment for which the graph was plotted. The x-axis of the graphs show the threshold (θ_j) and the y-axis show the attentional weight (w_j) for the jth logical dimension. The histogram at the top of each graph indicates the marginal posterior distribution for the threshold parameter (θ_j) and the histogram on the right indicates the marginal posterior distribution for the attentional weight parameter (w_j).

Table 2
Bayesian models used in this study.

Models	Description
Model I	Model I has four free parameters: c, α, θ_1 and θ_2. The prior probability distributions for these parameters is shown in Figure 1.
Model T_1	Model T_1 was constructed from model I by restricting the values that parameter θ_2 can take. The prior distribution of θ_2 was set to $DUniform([.82, .99])$. Model T_1 corresponds to the theoretical position that an FR dimension will be used only when it is learned with a high level ($> .82$) of accuracy.
Model T_0	Model T_0 was also constructed from model I by restricting the values that parameter θ_2 can take. The prior distribution of θ_2 was set to $DUniform([-.01, .16, .32, .49, .66])$. Model T_0 corresponds to the theoretical position that an FR dimension may be used even when it is not learned with a high level of accuracy.
Model T_{CA}	Model T_{CA} was constructed from model T_1 by setting parameter α to zero. Model T_{CA} corresponds to the theoretical position that participants used only the CA dimension to discriminate the two categories.

($\geq .82$) across the three experiments. This indicates that when participants used the jth dimension, their accuracy for the dimension was high (i.e. $t_{kj} > .82$).

Table 1 also shows the mean and standard deviation for parameters w_1 and w_2. The attentional weight for the CA dimension (w_1) was greater than the attentional weight for the FR dimensions (w_2) across the three experiments. This shows that participants gave more attention to the CA dimension compared to the FR dimensions across the three experiments.

Bayes factor analysis

We used Bayes factor analysis to compare different models that correspond to different theoretical positions. These models were constructed by restricting the values that the free parameters can take in model I (Figure 1). We compared two theoretical positions T_1 and T_0. Theoretical position T_1 states that an FR dimension will be used only when it is learned with a high level of accuracy (i.e. $t_{kj} > .82$). We modeled this theoretical position by letting the prior distribution for θ_2 to be $DUniform([.82, .99])$. Theoretical position T_0 states that an FR dimension may be used even when it is not learned with a high level of accuracy. Theoretical position T_0 is the null hypothesis for T_1 and was modeled by letting the prior distribution for θ_2 to be $DUniform([-.01, .16, .32, .49, .66])$. Note that in model T_0 we did not allow θ_2 to take the values .82 and .99. All the other details of models T_1 and T_0 were same as model I shown in Figure 1. Also, note that when $\theta_2 = -.01$ in model T_0, the FR dimension will always be used irrespective of the feature accuracy t_{kj}. This is because when $\theta_2 = -.01$, the value of the step function in Eqn. 2 will always be 1. So, model T_0 allows an FR dimension to be used at *any* level of accuracy, whereas for model T_1 the accuracy must be high ($t_{kj} > .82$).

Table 2 lists the models that we have used in this study. We used Bayes factor (Kass & Raftery, 1995) to compare model T_1 and model T_0. Bayes factor is the ratio of the marginal likelihood of the two models.

$$BF_{10} = \frac{P(Data|model\ T_1)}{P(Data|model\ T_0)} \quad (5)$$

Table 3

Log marginal likelihood for the models and the Bayes factors for the three experiments. The Bayes factors indicate extreme evidence in favour of model T_1.

	model T_1	model T_0	model T_{CA}	BF_{10}	BF_{1CA}
Exp. 1	-577.05	-608.67	-661.0	> 100	> 100
Exp. 2	-658.91	-668.52	-814.37	> 100	> 100
Exp. 3	-626.57	-685.36	-898.22	> 100	> 100

where $P(Data|model\ T_i)$ is the marginal likelihood for model T_i. The log marginal likelihood for the two models were estimated using the Bridge sampling method Gronau, Sarafoglou, et al. (2017); Gronau, Singmann, and Wagenmakers (2017).

For Experiment 1 data, the log marginal likelihood of model T_1 and model T_0 were found to be -577.05 and -608.67 respectively. The Bayes factor ($BF_{10} = e^{-577.05+608.67} > 100$, extreme evidence) showed that model T_1 provides a better explanation for the data compared to model T_0. Table 3 shows the log marginal likelihoods and the Bayes factor (BF_{10}) estimated for Experiments 2 and 3. The Bayes factor (BF_{10}) across all the three experiments were high (> 100, extreme evidence). Since model T_1 provides a better explanation for the data across the three experiments, it means that participants used the FR dimensions only when they had learned it with a high accuracy. In other words, the observed data provides evidence in favour of theoretical position T_1 compared to theoretical positions T_0 for all the three experiments.

The marginal posterior distribution of attentional weights showed that participants paid more attention to the CA dimension. So, we wanted to consider theoretical position T_{CA} which states that the observed data can be explained purely by the fact that participants were using the CA dimension to discriminate the categories. We constructed model T_{CA} by setting the attentional weights for the FR dimensions in model T_1 to zero. So, in model T_{CA} parameter α is always set to 0 (see Eqn. 3). All the other details of model T_{CA} are the same as that of model T_1. We have modified model T_1 to obtain model T_{CA} because we wanted to compare two models that differ in just one parameter.

For Experiment 1, the log marginal likelihood for model T_{CA} was found to be -661.0. The results of Bayes factor comparison between model T_1 and model T_{CA} shows that model T_1 provided a better explanation for the data in Experiment 1 ($BF_{1CA} = e^{-577.05+661.0} > 100$, extreme evidence). Table 3 shows the log marginal likelihoods and the Bayes factor (BF_{1CA}) estimated for Experiments 2 and 3. The results show that the Bayes factor was high across the three experiments. This shows that the observed data cannot be explained purely based on the CA strategy. Participants used the FR dimensions (non-minimal knowledge), but only when they had learned it with a high accuracy.

The conclusions drawn from the results of Bayesian modelling are consistent with the data shown in the summary table in the result section of the three experiments. The average number of FR dimensions learned with 100% accuracy (fourth row in the tables) is lowest for the $x < 2$ group and is highest for the $4 \leq x \leq 5$ group across the three experiments. In the groups shown in the tables, the number of stimuli dimensions used increases as more FR dimensions are learned with 100% accuracy.

Discussion

Our results show that in order for a stimuli dimension to be used for categorization it must be learned with a certain minimum accuracy across the three experiments. This minimum

accuracy (threshold) is on the higher side for both CA and FR dimensions. This means that the categorization behaviour cannot be explained purely based on the attention given to various dimensions; how accurately the stimuli dimensions were learned also determines the categorization strategy.

References

Brooks, S. P., & Gelman, A. (1998). General methods for monitoring convergence of iterative simulations. *Journal of computational and graphical statistics*, *7*(4), 434–455.

Gronau, Q. F., Sarafoglou, A., Matzke, D., Ly, A., Boehm, U., Marsman, M., ... Steingroever, H. (2017). A tutorial on bridge sampling. *Journal of mathematical psychology*, *81*, 80–97.

Gronau, Q. F., Singmann, H., & Wagenmakers, E.-J. (2017). bridgesampling: An r package for estimating normalizing constants. *arXiv preprint arXiv:1710.08162*.

Kass, R. E., & Raftery, A. E. (1995). Bayes factors. *Journal of the american statistical association*, *90*(430), 773–795.

Minda, J. P., & Smith, J. D. (2011). Prototype models of categorization: Basic formulation, predictions, and limitations. *Formal approaches in categorization*, 40–64.

Nosofsky, R. M. (2011). The generalized context model: An exemplar model of classification. *Formal approaches in categorization*, 18–39.

Plummer, M., et al. (2003). Jags: A program for analysis of bayesian graphical models using gibbs sampling. In *Proceedings of the 3rd international workshop on distributed statistical computing* (Vol. 124, pp. 1–10).

Su, Y.-S., & Yajima, M. (2012). R2jags: A package for running jags from r. *R package version 0.03-08, URL http://CRAN. R-project. org/package= R2jags*.

Thomas, S., & Srinivasan, N. (2022). Accurate knowledge about feature diagnosticities leads to less preference for unidimensional strategy. *Journal of Experimental Psychology: Learning, Memory, and Cognition*, *48*. doi: 10.1037/xlm0001151
